# Contemporary Analysis of VTE Risk After HPB Surgery: 2517 Consecutive Patients Treated With Extended Chemoprophylaxis

**DOI:** 10.1002/wjs.70407

**Published:** 2026-05-06

**Authors:** Anish J. Jain, Esther N. Dekker, Laura Prakash, Elsa M. Arvide, Yi‐Ju Chiang, Rebecca A. Snyder, Matthew H. G. Katz, Ching‐Wei D. Tzeng

**Affiliations:** ^1^ Department of Surgical Oncology University of Texas MD Anderson Cancer Center Houston Texas USA; ^2^ Department of Surgery Erasmus MC Cancer Institute Rotterdam the Netherlands

**Keywords:** anticoagulation, chemoprophylaxis, hepatopancreatobiliary surgery, transfusion, venous thromboembolism

## Abstract

Independent predictors of Venous Thromboembolism (VTE) within the first 45 post‐operative days on multivariate analyses.

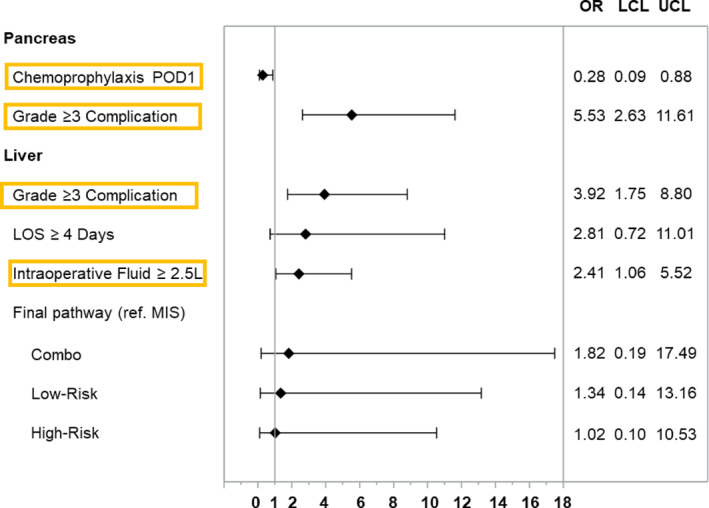

## 1 Introduction

At our institution, we iteratively improved postoperative outcomes of hepatopancreatobiliary (HPB) operations using risk‐stratified (e.g., risks of leaks and organ space infections) care pathways [1, 2]. One aspect that remains constant is our 28‐day (from surgery) discharge venous thromboembolism (VTE) chemoprophylaxis. However, this 28‐day “one size fits all” approach is based on broad clinical trials not specific to HPB surgery [3]. Thus, we sought to develop risk strata for discharge chemoprophylaxis by identifying patients at highest risk for postoperative VTE.

## 2 Methods

This was a retrospective cohort study of consecutive (non‐combination) hepatobiliary or pancreatic resections at MD Anderson Cancer Center, from January 2017 to April 2023. The study was approved by the institutional review board, with a waiver of informed consent (protocol PA18‐0856 and PA19‐0424). VTE prophylaxis consisted of pre‐incision subcutaneous heparin 5000 units, followed by an additional 5000 units in the recovery room six hours after closure. Starting the morning of postoperative day 1 (POD1), patients received enoxaparin 40 mg daily, continued after discharge to complete a total of 28 days of prophylaxis. The incidence of VTE events (deep venous thrombosis, pulmonary embolus, mesoportovenous thrombosis) and blood‐products transfusions were recorded. Factors associated with VTE occurring within 45 days after surgery (45d VTE) were compared. This timeframe was specifically chosen to exclude events potentially related to adjuvant therapy. Continuous variables were compared using the Mann–Whitney *U* test, and categorical variables were compared using the *χ* ^*2*^ test or Fisher's exact test, as appropriate. Univariate and Multivariate logistic analyses were used to identify factors associated with 45d VTE. The threshold for statistical significance was *p* < 0.05, with all tests 2‐sided. (SAS Enterprise Guide 8.3, SAS Inc.).

## 3 Results

Among 1001 pancreatectomies in 997 patients, 31 (3.1%) suffered 45d VTE, 73 (7.3%) received intraoperative transfusion, and 24 (2.4%) received postoperative transfusions.

A pancreatoduodenectomy was performed in 629 cases (62.8%), distal pancreatectomy in 344 cases (34.4%), central pancreatectomy in four cases (0.4%), total pancreatectomy in 17 cases (1.7%) and an enucleation in five cases (0.5%), and a completion pancreatectomy in two cases (0.2%).

Of the 31 patients who developed VTE after pancreatectomy, 12 (38.7%) had pancreatic ductal adenocarcinoma, one (3.2%) had bile duct adenocarcinoma, two (6.5%) had adenocarcinoma of the ampulla, one (3.2%) had pancreatic adenosquamous carcinoma, four (12.9%) had intraductal papillary mucinous neoplasm (IPMN), one (3.2%) had mucinous cystic neoplasm of the pancreas, eight (25.8%) had a pancreatic neuroendocrine tumor, one (3.2%) had a solid pseudopapillary tumor of the pancreas, and one (3.2%) had a pancreatic liposarcoma.

The VTE rate was 2.9% (15/513) in patients who received induction therapy and 3.3% (16/488) in those who did not (*p* = 0.86). The reasons for not administering POD1 prophylaxis in patients undergoing pancreatectomy were administration timing (e.g., administration on POD1 but not at correct pathway‐required time) in 17 patients, bleeding concerns in six patients, system‐related errors in three patients, abnormal laboratory values in seven patients, and clinician preference in six patients. No complications related to VTE prophylaxis were observed.

Among 1708 hepatectomies, 27 patients (1.6%) suffered 45d VTE with 28 events, 68 (4.0%) received intraoperative, and 109 (6.4%) received postoperative transfusions.

Of the 27 patients who developed VTE after hepatectomy, the indication for hepatectomy was colorectal liver metastases in 19 patients (70.4%), intrahepatic cholangiocarcinoma in one patient (3.7%), hepatocellular carcinoma in two patients (7.4%), adrenal cancer metastasis in one patient (3.7%), gallbladder carcinoma metastasis in one patient (3.7%), neuroendocrine tumor in the small bowel with liver metastasis in one patient (3.7%), retroperitoneal liposarcoma with metastasis in one patient (3.7%), and ovarian cancer metastasis in one patient (3.7%).

Pre‐incision, POD0, POD1, and discharge chemoprophylaxis administration rates were 94.6%, 56.8%, 95.8%, 91.9% for pancreatectomies, and 61%, 75.8%, 94%, 87.5% for hepatectomies.

**FIGURE 1:**
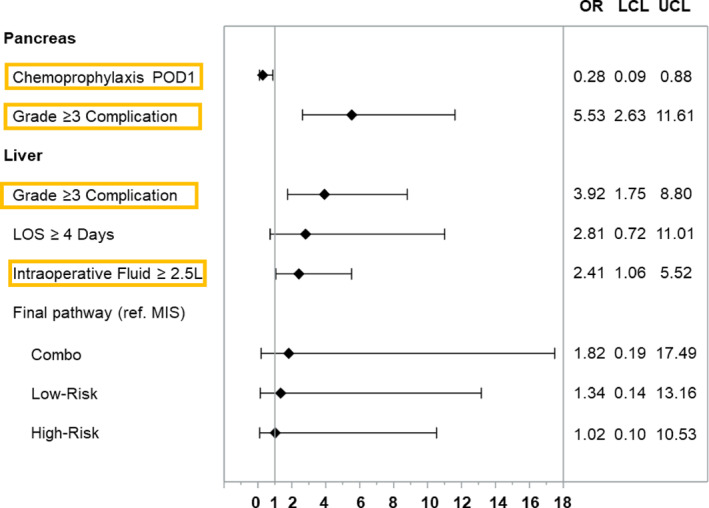
Independent predictors of Venous Thromboembolism (VTE) within the first 45 post‐operative days on multivariate analyses. Factors predictive of 45‐day VTE in pancreatectomy include major complications and adherence to perioperative chemoprophylaxis. Factors predictive of 45‐day VTE after hepatectomy include major complications and excessive intraoperative fluids. Final pathway represents institutional risk‐stratified postoperative care pathways, with MIS as the reference category. LCL, lower confidence limit; LOS, length of stay; MIS, minimally invasive surgery; OR, odds ratio; POD, postoperative day; UCL, upper confidence limit.

On multivariate analysis, predictors of post‐pancreatectomy VTE were ACCORDION [4] Grade ≥ III complications (OR 5.53; 95% CI 2.63–11.61; *p* < 0.01) and failure to receive chemoprophylaxis on POD1 (OR 3.57; 95% CI 1.13–11.1; *p* = 0.03). Risk factors for post‐hepatectomy VTE were Grade ≥ III complications (OR 3.92; 95% CI 1.75–8.80; *p* < 0.01) and intraoperative fluids ≥ 2.5 L (OR 2.41; 95% CI 1.06–5.52; *p* = 0.04) (Figure 1).

## 4 Discussion

Postoperative VTE risk can be mitigated to prevent downstream morbidity and mortality [5]. However, recent studies have highlighted the controversy of a universal recommendation for extended post‐discharge VTE chemoprophylaxis for HPB patients [6]. Lavikainen et al. conducted a systematic review and meta‐analysis, reporting procedure‐specific VTE risks in HPB surgeries, with rates as high as 6.4% for open distal pancreatectomy and 5.3% for major open liver resection, highlighting the variability of VTE risk across different procedures [7]. Real‐world studies have shown poor compliance with recommended guidelines despite the known risks of VTE [8]. In this study, we identified adherence to early POD1 chemoprophylaxis as a controllable variable associated with decreased VTE incidence.

We also identified major (i.e., requiring interventions) complications as potentially targetable risk factors for VTE. These patients may need extended chemoprophylaxis, especially considering complications of HPB operations (e.g., leaks and organ space infections) can persist beyond the standard 28 days of chemoprophylaxis.

Study limitations include the single‐institutional retrospective design of the study, which may not reflect the real‐world compliance for chemoprophylaxis prescriptions. Additionally, we did not have data on patient compliance for the full 28‐day course or a systematic approach for preoperative or postoperative asymptomatic VTE screening. However, this study consists of prospectively validated (by faculty and advanced practice providers biweekly) data that accurately conveys chemoprophylaxis administration rates, perioperative complications, and transfusion rates in a large cohort of HPB patients treated with enhanced recovery pathways.

In order to increase adherence to discharge chemoprophylaxis and potentially improve patient comfort, a personalized approach could be prospectively studied using extended chemoprophylaxis for HPB patients with dynamically graded (as their postoperative course develops) high‐risk factors (e.g., grade ≥ III complications or missing POD1 chemoprophylaxis) versus de‐escalated chemoprophylaxis in low‐risk patients after a shorter universal course.

In conclusion, risk‐stratified approaches for postoperative chemoprophylaxis warrant prospective investigation and possible incorporation into postoperative care pathways for HPB patients.

## Author Contributions

**Anish J. Jain:** conceptualization, writing – original draft, visualization. **Esther N. Dekker:** writing – review and editing. **Laura Prakash:** writing – review and editing, conceptualization, investigation. **Elsa M. Arvide:** investigation, conceptualization, writing – review and editing. **Yi‐Ju Chiang:** writing – review and editing, formal analysis, data curation, supervision, validation, methodology, visualization. **Rebecca A. Snyder:** writing – review and editing, supervision, methodology. **Matthew H.G. Katz:** conceptualization, investigation, validation, supervision, writing – review and editing, methodology. **Ching‐Wei D. Tzeng:** conceptualization, writing – original draft, validation, writing – review and editing, supervision, methodology.

## Funding

Dr. Tzeng is supported in part by the University Cancer Foundation and the Duncan Family Institute for Cancer Prevention and Risk Assessment via a Cancer Survivorship Research Seed Money Grant at the University of Texas MD Anderson Cancer Center and an Andrew Sabin Family Foundation Fellowship. Dr Tzeng reported receiving consultant fees and a sponsored research agreement from PanTher outside the submitted work.

## Conflicts of Interest

The authors declare no conflicts of interest.

## Data Availability

The data that support the findings of this study are available from the corresponding author upon reasonable request.
